# QTL epistasis plays a role of homeostasis on heading date in rice

**DOI:** 10.1038/s41598-023-50786-x

**Published:** 2024-01-03

**Authors:** Lilong Huang, Jichun Tang, Bihuang Zhu, Guodong Chen, Leyi Chen, Suhong Bu, Haitao Zhu, Zupei Liu, Zhan Li, Lijun Meng, Guifu Liu, Shaokui Wang

**Affiliations:** 1 Guangdong Key Laboratory of Plant Molecular Breeding, South China Agricultural University, Guangzhou, 510642 People’s Republic of China; 2 Kunpeng Institute of Modern Agriculture at Foshan, Foshan, 528200 People’s Republic of China

**Keywords:** Genetics, Quantitative trait

## Abstract

If there was no gene interaction, the gene aggregation effect would increase infinitely with the increase of gene number. Epistasis avoids the endless accumulation of gene effects, playing a role of homeostasis. To confirm the role, QTL epistases were analyzed by four single-segment substitution lines with heading date QTLs in this paper. We found that QTLs of three positive effects and one negative effect generated 62.5% negative dual QTL epistatic effects and 57.7% positive triple QTL epistatic effects, forming the relationship “positive QTLs-negative one order interactions-positive two order interactions”. In this way, the aggregation effect of QTLs was partially neutralized by the opposite epistatic effect sum. There also were two exceptions, QTL *OsMADS50* and gene *Hd3a-2* were always with consistent effect directions with their epistases, implying they could be employed in pyramiding breeding with different objectives. This study elucidated the mechanism of epistatic interactions among four QTLs and provided valuable genetic resources for improving heading date in rice.

## Introduction

High yield is the eternal theme of rice breeding. Heading date in rice is one of the important agronomic traits, which is closely related with yield^1,2^. Heading date (anthesis) is the critical mark of the transition from vegetative growth to reproductive growth^3,4^. The length of heading date will affect the accumulation of photosynthetic products, then the grain filling process and finally the yield^5,6^. Heading date also determines the adaptation for current varieties of cultivated rice to specific cropping locations and growing seasons, playing an important role for producing and introducing of rice varieties^7,8^. In practice, the conflict ‘early ripening and high yield’ and ‘late ripening and adversity’ exists always^9–11^. Thus it is of great theoretical and practical significance to study the molecular regulation mechanism of rice heading date for molecular breeding and agricultural production.

Heading date in rice is also a complex quantitative trait, regulated by a multiple QTL system companying with additive, dominance and epistasis, as well as their interaction with environments^7,12^. Researches over the last 20 years have found that there are at least 734 QTLs on heading date according to the data published on Gramene website (http://www.gramene.org/qtl/). There are two independent flowering pathways to control heading date in rice, one is the conserved *Hd1*-dependent pathway and the other unique *Ehd1*-dependent^13,14^. *Hd3a* is located in the center of two pathways, which encodes directly florigen to form flowers and is always regulated by the upstream QTLs of *Hd1*, *Ehd1*, *OsMADS50* and others^12^. *Hd1* upregulates *Hd3a* under short day and downregulates it under long day^15,16^, while *Ehd1* and *OsMADS50* always accelerate heading under both short and long days by upregulating *Hd3a*^4,17^. However, recent studies revealed more complex rice-specific gene networks regulating the heading^4,12^. The regulating relationship and the regulating degree still need further discussion.

Gene interactions, including allelic interactions (dominance) and nonallelic interactions (epistasis), play an important role in the flowering of rice^18,19^. Epistasis is one of important genetic components for a complex quantitative trait, which was defined as the effect of one gene modified by another gene or several other genes (biological epistasis) and is estimated as the deviation from additivity in a linear statistical model (statistical epistasis)^20,21^. To date studies have targeted mostly on biological epistasis by molecular means, while few on statistical epistasis since the limitations of research materials and statistical methods^11,15,22^. Using QTL near isogenic lines or single segment substitution lines (SSSLs) dual QTL epistatic effects were estimated effectively on lots of important quantitative traits^18,23–25^. However, the interaction among multiple QTLs, called as “high-order epistasis”^20,21^, was reported rarely. Higher-order epistasis is even more common in the multiple gene system and more important to keep homeostasis of organism^21,26,27^.

In this paper, four SSSLs, which were confirmed with heading date QTLs in our previous study^28^, were applied for estimation of epistatic effects among QTLs. Some crossing combinations of dual QTLs and triple QTLs were configured to analyze QTL genetic effects, including single QTL effects (additive and dominance) and epistatic effects (dual QTL interaction and triple QTL interaction). The trial was conducted at three seasons in two years so that the environmental sensitivity for these genetic components could be evaluated. This paper was with two purposes, one was to quantitatively analyze the interaction mechanism for the four heading date QTLs in order to confirm the role of epistases, and the other was to excavate the favorable gene materials for design breeding on heading date in rice.

## Results

### Phenotypic variation on HD

The phenotypic values on HD were mainly influenced by environments, genotypes, genotypes × environments, and experimental error. The environments *e1* and *e3* represented the short day condition, which shorten the heading periods. While the *e2* was the long day condition, delaying flowering. These genotypes, involving in four QTLs, included 65 different types. The average HD of the genotypes ranged from 72.9d to 105.6d, with the standard deviation of 8.9d (data not shown). Joint analysis of variance on phenotypic values of HD in the three environments showed that the mean square of interaction between genotypes and environments was also significant at *p* = 0.0001 (Supplementary Table S1). According to the equivalence relations between the expecting mean squares (EMSs) and the mean squares, the variance components could be estimated, and then the general heritability (expressed in all environments) and the peculiar heritability (expressed in special environments only) were estimated as 31.78% and 1.27% for HD, respectively. The results verified that HD was a complex trait, which was simultaneously controlled by genetic factors, environmental factors, and their interactions etc., just the special heritability being small.

### Additive effects (a) and additive × environment interaction effects (ae) of QTLs on HD

Genotype is composed of genes, thus genotypic effect can be divided into gene effects. Gene effects generally include additive, dominance, epistasis and their interaction effects with environments. SSSLs and their pyramiding materials allow estimation of gene effects. Additive effects (*a*) and additive × environment interaction effects (*ae*) of QTLs on HD were estimated by the difference values between homozygotes of SSSLs and receptor HJX74 (Table 1). *Hd1* hadn’t significant additive. *Ehd1* had significant additive to delay heading date only in the *e3* environment. *OsMADS50* shorten heading date of 6.1d by the additive, but there were significant difference among different environments. Hd3a increased heading date of 5.6d, which wasn’t influenced by environments.

**Table 1:** Additive effects (*a*) and additive × environment interaction effects (*ae*) of QTLs on heading date (day, d).

QTL	a	ae1	ae2	ae3
*Ehd1*				2.1*
*OsMADS50*	–6.1**	–1.9*		
*Hd3a*	5.6**			
*Hd1*				

“–” Indicated that the allele from the donor shorten heading date. “*” and “**” represented the significance at the probability levels 0.05 and 0.01, respectively.

In summary, two QTLs of *OsMADS50* and *Hd3a* had general additives, which could be detected in different environments. *OsMADS50* promoted flowering and was regulated by environments, while *Hd3a* inhibited heading. *Ehd1* was a specific QTL, which be expressed only in specific environments. *Hd1* was detected without additive.

### Dominant effects (d) and dominance × environment interaction effects (de) of QTLs on HD

Dominance is the interaction between alleles. Dominant effects (*d*) and dominance × environment interaction effects (*de*) of QTLs were estimated on HD by the difference values between heterozygotes of SSSLs and receptor HJX74 (Table 2). All of the four QTLs were detected with significant dominant effects. *OsMADS50* shorten HD of 8.0d, being a early ripe gene. While *Hd3a* delayed HD 18.5d, a very late maturation gene. Both *Ehd1* and *Hd3a* were environmental sensitive, and the others were environmental stable. Comparing with the additives, the dominances of the QTLs had consistent effect directions and larger effect values. For instance, the dominant degree (*d/a*) of *Ehd1* equaled to 2.07, being larger than 1. The results indicated that all of the four QTLs were super-dominant loci.

**Table 2:** Dominant effects (*d*) and dominance × environment interaction effects (*de*) of QTLs on heading date (day, d).

QTL	d	de1	de2	de3
*Ehd1*	2.7**	− 2.3*		2.6*
*OsMADS50*	− 8.0**			
*Hd3a*	18.5**		− 2.4*	
*Hd1*	1.7*			

“–” Indicated that the allele from the donor shorten heading date. “*” and “**” represented the significance at the probability levels 0.05 and 0.01, respectively.

In summary, all of four QTLs were with the genes associated with heading date. Three QTLs carried with additive and dominance simultaneously, while *Hd1* with dominance only. Except for *Hd1*, additives or dominances of all QTLs were influenced by environments. All of these QTLs could be applied in heterosis for the target of early ripening or late ripening.

### Pyramiding effects of QTLs (g) and their interaction effects with environments (ge) on HD

After the effects of single QTL were tested, we conducted the polymerization of dual-QTLs and triple-QTLs to test the pyramiding effects. The pyramiding effects were estimated by the difference values between the pyramiding materials and HJX74 (Table 3). Of the 56 pyramiding materials measured, there were 49 estimations to reach the significance level of p < 0.05 and 4 pyramiding effects to be significant only in special environments. Three combinations hadn’t significant pyramiding effects. Since *OsMADS50* carried with large negative effects (additive or dominance), most combinations with *OsMADS50* appeared negative pyramiding effects, indicating that the QTL had strong expression power to promote heading. *Hd3a* had large positive effects, thus the pyramiding effects of *Hd3a*, especially being the homozygote *Hd3a-2*, were always with large positive effects to delay flowering. When *OsMADS50* encountering *Hd3a-1*, the pyramiding effects were usually negative. While the combinations between *OsMADS50* and *Hd3a-2* generated always positive pyramiding effects. Thus *OsMADS50* was suggested to be applied to early ripe breeding, while *Hd3a-2* to late ripe breeding.

**Table 3:** Pyramiding effects of QTLs and their interaction effects with environments on heading date (day, d).

QTL combination			g	ge1	ge2	ge3
*Ehd1-1*	*OsMADS50-1*		− 6.8**		3.2*	
*Ehd1-1*	*OsMADS50-2*		− 6.6**			
*Ehd1-1*	*Hd3a-1*		6.0**			3.2*
*Ehd1-1*	*Hd3a-2*		21.3**			
*Ehd1-1*	*Hd1-1*					
*Ehd1-1*	*Hd1-2*		1.7*	− 2.9*		3.2*
*Ehd1-2*	*OsMADS50-1*		− 3.9**	− 3.4**		
*Ehd1-2*	*OsMADS50-2*		− 5.9**	− 2.6*		
*Ehd1-2*	*Hd3a-1*		7.6**	− 2.8*		2.5*
*Ehd1-2*	*Hd3a-2*		23.2**	− 4.4**	2.5*	
*Ehd1-2*	*Hd1-1*					
*Ehd1-2*	*Hd1-2*		2.7**	− 2.9*		2.6*
*OsMADS50-1*	*Hd3a-1*		− 3.0**			
*OsMADS50-1*	*Hd3a-2*		4.8**			
*OsMADS50-1*	*Hd1-1*		− 6.6**			
*OsMADS50-1*	*Hd1-2*		− 7.6**		2.6*	
*OsMADS50-2*	*Hd3a-1*		− 6.2**			
*OsMADS50-2*	*Hd3a-2*			3.6**	− 2.8*	
*OsMADS50-2*	*Hd1-1*		− 9.0**			
*OsMADS50-2*	*Hd1-2*		− 9.5**			
*Hd3a-1*	*Hd1-1*		4.9**			
*Hd3a-1*	*Hd1-2*		5.7**			
*Hd3a-2*	*Hd1-1*		16.4**	2.7*		− 4.9**
*Hd3a-2*	*Hd1-2*		13.1**	5.7**		− 7.9**
*Ehd1-1*	*OsMADS50-1*	*Hd3a-1*		− 5.6**		6.0**
*Ehd1-1*	*OsMADS50-1*	*Hd3a-2*	9.4**	− 3.1*		3.1*
*Ehd1-1*	*OsMADS50-1*	*Hd1-1*	− 6.6**			
*Ehd1-1*	*OsMADS50-1*	*Hd1-2*	− 6.5**		2.5*	
*Ehd1-1*	*OsMADS50-2*	*Hd3a-1*	− 2.9**			
*Ehd1-1*	*OsMADS50-2*	*Hd3a-2*	3.9**			
*Ehd1-1*	*OsMADS50-2*	*Hd1-1*	− 8.2**			
*Ehd1-1*	*OsMADS50-2*	*Hd1-2*	− 8.7**			
*Ehd1-1*	*Hd3a-1*	*Hd1-1*	5.3**			
*Ehd1-1*	*Hd3a-1*	*Hd1-2*	5.7**			
*Ehd1-1*	*Hd3a-2*	*Hd1-1*	19.9**			
*Ehd1-1*	*Hd3a-2*	*Hd1-2*	19.4**			
*Ehd1-2*	*OsMADS50-1*	*Hd3a-1*		− 4.7**	2.5*	
*Ehd1-2*	*OsMADS50-1*	*Hd3a-2*	12.5**	− 4.2**		2.9*
*Ehd1-2*	*OsMADS50-1*	*Hd1-1*	− 5.2**		3.0*	
*Ehd1-2*	*OsMADS50-1*	*Hd1-2*	− 3.8**			
*Ehd1-2*	*OsMADS50-2*	*Hd3a-1*	− 1.9*			
*Ehd1-2*	*OsMADS50-2*	*Hd3a-2*	8.4**	− 3.1*		
*Ehd1-2*	*OsMADS50-2*	*Hd1-1*	− 6.2**			
*Ehd1-2*	*OsMADS50-2*	*Hd1-2*	− 6.6**		3.0*	
*Ehd1-2*	*Hd3a-1*	*Hd1-1*	8.7**	− 2.9*		2.6*
*Ehd1-2*	*Hd3a-1*	*Hd1-2*	7.1**			
*Ehd1-2*	*Hd3a-2*	*Hd1-1*	21.9**			
*Ehd1-2*	*Hd3a-2*	*Hd1-2*	22.9**	− 2.5*		
*OsMADS50-1*	*Hd3a-1*	*Hd1-1*	− 3.8**			
*OsMADS50-1*	*Hd3a-1*	*Hd1-2*	− 2.8**		2.7*	
*OsMADS50-1*	*Hd3a-2*	*Hd1-1*	2.9**			
*OsMADS50-1*	*Hd3a-2*	*Hd1-2*	4.7**			
*OsMADS50-2*	*Hd3a-1*	*Hd1-1*	− 4.4**			
*OsMADS50-2*	*Hd3a-1*	*Hd1-2*	− 5.2**			
*OsMADS50-2*	*Hd3a-2*	*Hd1-1*		4.0**		− 2.6*
*OsMADS50-2*	*Hd3a-2*	*Hd1-2*				

*g* was pyramiding effect of QTL combination, and *ge* was their interaction effect with environment. *QTL-1* and *QTL-2* respectively represented the heterozygote and homozygote of the QTL. “–” indicated that the allele from the donor shorten heading date. “*” and “**” represented the significance at the probability levels 0.05 and 0.01, respectively.

### The network relationship among the 4 QTLs

Analysis of pyramiding effects of QTLs contributed to reveal the promoting and inhibiting relationship between QTLs. When the effect of one QTL remains unchanged under the background of another QTL, the two QTLs are independent each other. Otherwise, one QTL is promoted or inhibited by another QTL when the QTL effect changes. The effect changes of the 4 QTLs showed in Supplementary Fig. S1. For QTL *Ehd1*, QTLs *OsMADS50* and *Hd3a* apparently reduced and increased its effect respectively, while QTL *Hd1* changed it few. Thus it was suggested that *Hd1* was independent of *Ehd1*, while *OsMADS50* and *Hd3a* inhibited and promoted *Ehd1*, respectively. However, the regulations for these QTLs to *Ehd1* were influenced by environments, existing the difference across environments. For gene *OsMADS50-1*, gene *Ehd1-2* and QTL *Hd3a* had the significant effects to inhibit its expression, while QTL *Hd1* changed it few. For gene *OsMADS50-2*, gene *Hd3a-2* still inhibited it, while *Hd1* promoted its expression. All of QTLs inhibited *Hd3a-1* and promoted *Hd3a-2* except for *OsMADS50* inhibiting slightly the expression of *Hd3a-2*. *OsMADS50* and *Hd3a* inhibited and promoted *Hd1* respectively, while *Ehd1* affected *Hd1* few.

**Figure 1:**
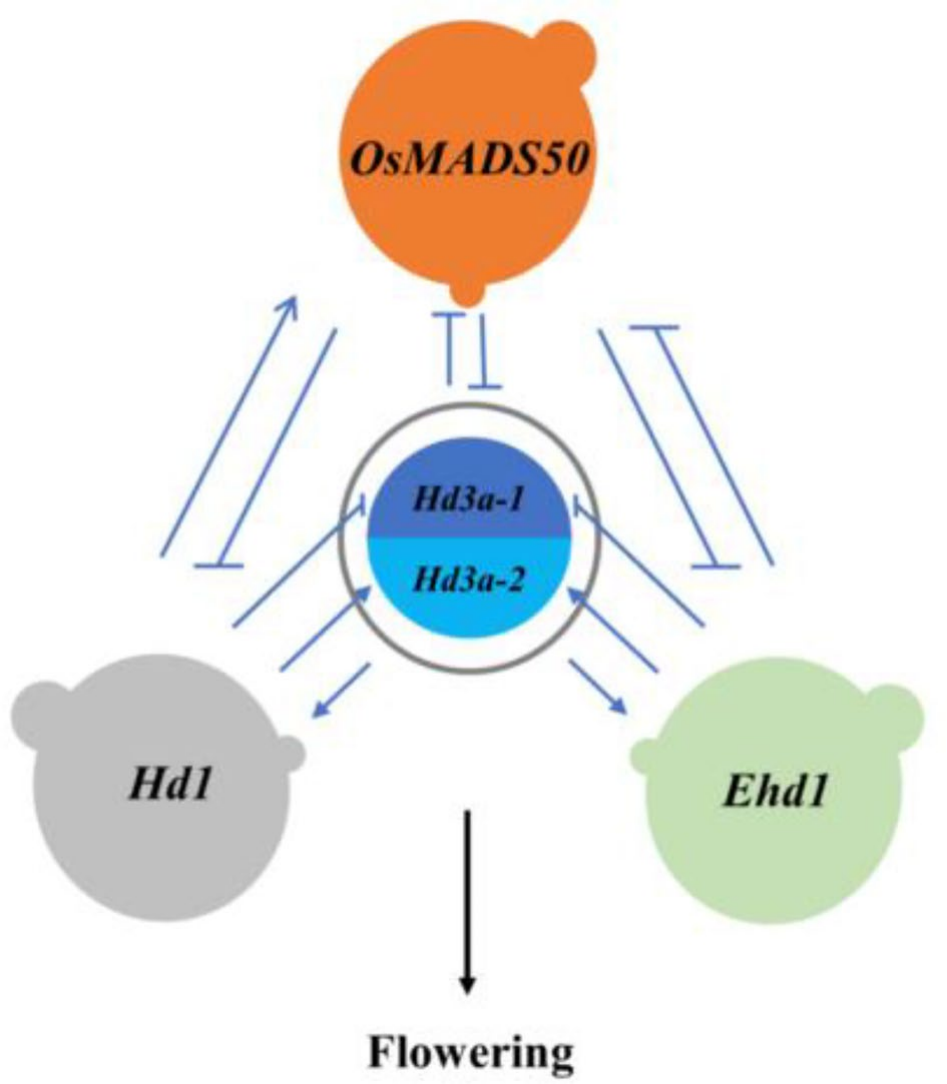
The network relationship among the four QTLs on heading date in rice. *Hd3a-1* and *Hd3a-2* were the heterozygote and homozygote on *Hd3a*, respectively. → promoting; $\dashv$ inhibiting. Except that *Hd1* and *Ehd1* were independent, the other QTLs were related to each other, promoting or inhibiting.

In summary, *Hd1* and *Ehd1* were independent, while the other QTLs were related to each other, promoting **or inhibiting (Fig. 1). We could clearly see that at least four flowering paths since *Hd1* and *Ehd1* were independent each other. They regulated flowering via to directly regulate *Hd3a* or to indirectly influence *OsMADS50*, respectively. *Hd3a* is the induce factor of flowering, which is regulated by lots of upstream or downstream QTLs.

### Epistatic effects (e) and epistasis × environment interaction effects (ee) of QTLs on HD

#### The epistatic effects and epistasis × environment interaction effects between dual QTLs

Epistasis is the interaction among nonalleles. Based on SSSLs and their pyramiding materials, the epistatic effects and epistasis×environment interaction effects between dual QTLs were estimated by the residual effects between the pyramiding effects and the sum of single QTL effects (Table 4). Here epistatic effects estimated included four interaction components such as dominance-dominance, dominance-additive, additive- dominance and additive-additive, in proper order corresponding *QTL-1--QTL-1, QTL-1--QTL-2, QTL-2--QTL-1* and *QTL-2--QTL-2* in Table.

**Table 4:** The epistatic effect (*e*) and epistasis × environment interaction effects (*ee*) between dual QTLs on HD (day, d).

QTL		*e*	*ee1*	*ee2*	*ee3*
*Ehd1-1*	*OsMADS50-1*			3.5*	− 3.4*
*Ehd1-2*	*OsMADS50-1*	2.7**			
*Ehd1-1*	*OsMADS50-2*	− 3.2**			
*Ehd1-2*	*OsMADS50-2*				
*Ehd1-1*	*Hd3a-1*	− 15.2**			
*Ehd1-2*	*Hd3a-1*	− 12.2**		3.7*	
*Ehd1-1*	*Hd3a-2*	13.0**			-3.5*
*Ehd1-2*	*Hd3a-2*	16.2**		4.1*	
*Ehd1-1*	*Hd1-1*	− 3.4**	3.3*		-3.1*
*Ehd1-2*	*Hd1-1*				
*Ehd1-1*	*Hd1-2*				
*Ehd1-2*	*Hd1-2*				
*OsMADS50-1*	*Hd3a-1*	− 13.5**		3.5*	
*OsMADS50-2*	*Hd3a-1*	− 18.6**			
*OsMADS50-1*	*Hd3a-2*	7.2**			
*OsMADS50-2*	*Hd3a-2*		6.4**	− 3.6*	
*OsMADS50-1*	*Hd1-1*				
*OsMADS50-2*	*Hd1-1*	− 4.6**			
*OsMADS50-1*	*Hd1-2*	− 7.6**			
*OsMADS50-2*	*Hd1-2*	− 4.4**			
*Hd3a-1*	*Hd1-1*	− 15.3**			
*Hd3a-2*	*Hd1-1*	9.1**	5.0**		− 7.1**
*Hd3a-1*	*Hd1-2*				
*Hd3a-2*	*Hd1-2*	6.6**	7.3**		− 9.7**

The numbers 1 and 2 immediately following the QTL names indicated the heterozygotes and the homozygotes of QTLs, respectively. For instance, *Ehd1-1* and *Ehd1-2* were the heterozygote and the homozygote of *Ehd1*, respectively. *ee1*, *ee2* and *ee3* represented the interaction effects of epistasis and three environments, respectively. “–” indicated that the interaction between the alleles from the donor shorten heading date. “*” and “**” represented the significance at the probability levels 0.05 and 0.01, respectively.

All of six pairs of QTLs were detected with significant epistatic effects, further confirming the prevalence of epistatic interactions among QTLs on heading date. One QTL interacted usually with the other three QTLs. Of 24 epistatic components, 18 estimations reached the significant level of *p*<0.05 or 0.01. Where 9 epistatic components were environmentally sensitive, which accompanied with significant epistasis×environments. Two pairs of genes, *Ehd1-1* and *OsMADS50-1*, *OsMADS50-2* and *Hd3a-2*, showed significant epistatic interactions in particular environments only. Eshed and Zamir^24^ found first the phenomena less than-additive epistatic interactions between QTLs in tomato. This paper found also that 10 estimations were negative, occupying up 62.5% of 16 significant epistatic components. Generally, negative epistasis is mainly derived from the interaction of positive QTLs^28,29^. Here three QTLs, *Ehd1*, *Hd3a* and *Hd1*, carried positive effects, so their epistases appeared mostly negative effects. An interesting result was that *Hd3a-1* and *Hd3a-2* always generated large, opposite epistases, i.e. *Hd3a-1* was mostly with negative epistatic effects while *Hd3a-2* with positive (Table 4). Another result was that *OsMADS50*, with large and negative additive or dominance effects, mostly generated negative epistases. The genetic mechanisms for these two results need to be further explored. The results also indicated that *OsMADS50* and *Hd3a-2* could be applied to different objectives of ripe breeding.

#### The epistatic effects and epistasis × environment interaction effects among triple QTLs

In the multiple gene genetic system, the interactions among multiple genes are inevitable. The epistatic effect and epistasis×environment interaction effects among triple QTLs were estimated by the residual effect between the pyramiding effect and the sum of single QTL effects and the interaction effects between dual QTLs (Table 5).

**Table 5:** The epistatic effect (*e*) and epistasis × environment interaction effects (*ee*) among triple QTLs on HD (day, d).

QTL			e	ee1	ee2	ee3
*Ehd1-1*	*OsMAD50-1*	*Hd3a-1*	16.8**		− 5.6*	7.5**
*Ehd1-1*	*OsMAD50-1*	*Hd3a-2*	− 9.7**			7.4**
*Ehd1-1*	*OsMAD50-1*	*Hd1-1*				
*Ehd1-1*	*OsMAD50-1*	*Hd1-2*	8.9**			
*Ehd1-1*	*OsMAD50-2*	*Hd3a-1*	19.0**			
*Ehd1-1*	*OsMAD50-2*	*Hd3a-2*	− 9.3**	− 6.0*		
*Ehd1-1*	*OsMAD50-2*	*Hd1-1*	4.8**			
*Ehd1-1*	*OsMAD50-2*	*Hd1-2*	3.3*			
*Ehd1-1*	*Hd3a-1*	*Hd1-1*	16.3**			
*Ehd1-1*	*Hd3a-1*	*Hd1-2*				
*Ehd1-1*	*Hd3a-2*	*Hd1-1*	− 8.8**	− 5.9*		7.0**
*Ehd1-1*	*Hd3a-2*	*Hd1-2*	− 7.5**	− 5.7*		9.0**
*Ehd1-2*	*OsMAD50-1*	*Hd3a-1*	11.1**			
*Ehd1-2*	*OsMAD50-1*	*Hd3a-2*	− 12.6**			
*Ehd1-2*	*OsMAD50-1*	*Hd1-1*				
*Ehd1-2*	*OsMAD50-1*	*Hd1-2*	6.4**			
*Ehd1-2*	*OsMAD50-2*	*Hd3a-1*	14.0**			
*Ehd1-2*	*OsMAD50-2*	*Hd3a-2*	− 8.7**			
*Ehd1-2*	*OsMAD50-2*	*Hd1-1*	4.5**			
*Ehd1-2*	*OsMAD50-2*	*Hd1-2*				
*Ehd1-2*	*Hd3a-1*	*Hd1-1*	16.6**			
*Ehd1-2*	*Hd3a-1*	*Hd1-2*				
*Ehd1-2*	*Hd3a-2*	*Hd1-1*	− 10.1**		− 5.5*	6.8**
*Ehd1-2*	*Hd3a-2*	*Hd1-2*	− 8.2**		− 5.3*	9.0**
*OsMAD50-1*	*Hd3a-1*	*Hd1-1*	13.1**			
*OsMAD50-1*	*Hd3a-1*	*Hd1-2*	8.6**			
*OsMAD50-1*	*Hd3a-2*	*Hd1-1*	− 12.5**			
*OsMAD50-1*	*Hd3a-2*	*Hd1-2*		− 7.0**		10.7**
*OsMAD50-2*	*Hd3a-1*	*Hd1-1*	20.0**			
*OsMAD50-2*	*Hd3a-1*	*Hd1-2*	6.1**			
*OsMAD50-2*	*Hd3a-2*	*Hd1-1*	-6.0**	− 5.1*		5.7*
*OsMAD50-2*	*Hd3a-2*	*Hd1-2*	-4.2**	− 10.4**		12.6**

The numbers 1 and 2 immediately following the QTL names indicated the heterozygotes and the homozygotes of QTLs, respectively. *ee1*, *ee2* and *ee3* represented the interaction effects of epistasis and three environments, respectively. “–” indicated that the interaction between the alleles from the donor shorten heading date. “*” and “**” represented the significance at the probability level 0.05 and 0.01, respectively.

Of 32 epistatic effects, 81.25% estimations were statistically significant, also indicating the prevalence of epistasis. Where 10 epistatic components were environmentally sensitive, which accompanied with significant epistasis×environment interaction effects. While the combination of *OsMADS50*/*Hd3a-2*/*Hd1-2* showed significant epistatic interactions in particular environments only. However, most of epistatic effects among triple QTLs were positive, occupying up 57.7% of 26 significant estimations. The pattern “positive effects of single QTLs-negative epistatic effects between dual QTLs-positive epistatic effects among triple QTLs” was perhaps a trend, but it needs to be further verified. *Hd3a-1* and *Hd3a-2* always generated large, inverse epistases in triple QTL interactions also. On magnitude, the average of epistatic effects among three QTLs was approximately 8.6±5.5d, while that between dual QTLs was 6.8±5.9d. It was showed that triple QTL interactions might play a more important role than dual QTL interactions.

In fact, epistasis in a three QTL genotype includes each of two QTL interactions and three QTL interaction, called mixed epistasis. The mixed epistatic effect (*e*) and mixed epistasis×environment interaction effects (*ee*) among triple QTLs were estimated by the residual effect between the pyramiding effect (Table 3) and the sum of single QTL effects (Table 1 and Table 2). The estimations were listed in Table 6.

**Table 6:** The mixed epistatic effect (*e*) and mixed epistasis × environment interaction effects (*ee*) among triple QTLs on HD (day, d).

QTL			*e*	*ee1*	*ee2*	*ee3*
*Ehd1-1*	*OsMADS50-1*	*Hd3a-1*	− 13.4**	− 4.5*		
*Ehd1-1*	*OsMADS50-1*	*Hd3a-2*	19.1**			
*Ehd1-1*	*OsMADS50-1*	*Hd1-1*	− 3.0*			
*Ehd1-1*	*OsMADS50-1*	*Hd1-2*				
*Ehd1-1*	*OsMADS50-2*	*Hd3a-1*	− 18.0**			
*Ehd1-1*	*OsMADS50-2*	*Hd3a-2*	− 5.3**			
*Ehd1-1*	*OsMADS50-2*	*Hd1-1*	− 3.8**			
*Ehd1-1*	*OsMADS50-2*	*Hd1-2*	− 6.2**	4.8*		− 5.3*
*Ehd1-1*	*Hd3a-1*	*Hd1-1*	− 17.6**			− 4.8*
*Ehd1-1*	*Hd3a-1*	*Hd1-2*	-16.4**			
*Ehd1-1*	*Hd3a-2*	*Hd1-1*	9.9**	4.4*		− 6.7**
*Ehd1-1*	*Hd3a-2*	*Hd1-2*	10.2**			
*Ehd1-2*	*OsMADS50-1*	*Hd3a-1*	− 11.8**	− 4.8*	5.9*	
*Ehd1-2*	*OsMADS50-1*	*Hd3a-2*	13.6**			
*Ehd1-2*	*OsMADS50-1*	*Hd1-1*				
*Ehd1-2*	*OsMADS50-1*	*Hd1-2*				
*Ehd1-2*	*OsMADS50-2*	*Hd3a-1*	− 15.6**			
*Ehd1-2*	*OsMADS50-2*	*Hd3a-2*	7.6**			
*Ehd1-2*	*OsMADS50-2*	*Hd1-1*	− 3.1*			
*Ehd1-2*	*OsMADS50-2*	*Hd1-2*	− 2.7**			− 4.8*
*Ehd1-2*	*Hd3a-1*	*Hd1-1*	− 12.8**			
*Ehd1-2*	*Hd3a-1*	*Hd1-2*	− 13.6**			
*Ehd1-2*	*Hd3a-2*	*Hd1-1*	13.3**			− 4.6*
*Ehd1-2*	*Hd3a-2*	*Hd1-2*	15.1**			
*OsMADS50-1*	*Hd3a-1*	*Hd1-1*	− 16.0**			
*OsMADS50-1*	*Hd3a-1*	*Hd1-2*	− 14.2**		4.4*	
*OsMADS50-1*	*Hd3a-2*	*Hd1-1*	3.6**			− 4.4*
*OsMADS50-1*	*Hd3a-2*	*Hd1-2*	6.2**			
*OsMADS50-2*	*Hd3a-1*	*Hd1-1*	− 18.5**			
*OsMADS50-2*	*Hd3a-1*	*Hd1-2*	− 18.5**			
*OsMADS50-2*	*Hd3a-2*	*Hd1-1*		8.2**		− 5.3*
*OsMADS50-2*	*Hd3a-2*	*Hd1-2*		4.2*		

The numbers 1 and 2 immediately following the QTL names indicated the heterozygotes and the homozygotes of QTLs, respectively. *ee1*, *ee2* and *ee3* represented the interaction effects of mixed epistasis and three environments, respectively. “–” indicated that the interaction between the alleles from the donor shorten heading date. “*” and “**” represented the significance at the probability levels 0.05 and 0.01, respectively.

90.6% (out of 32) mixed epistases reached statistically significant levels, 11 estimations of which were influenced by environments. Two combinations, *OsMADS50*/*Hd3a-2*/*Hd1-1* and *OsMADS50*/*Hd3a-2*/*Hd1-2*, showed significant epistatic interactions in particular environments only. 66.7% of 27 significant epistatic effects were negative, acting as a balance role of single QTL effects on the whole. Similarly, *Hd3a-1* and *Hd3a-2* generated also opposite mixed epistases in QTL interactions. On magnitude, the average of mixed epistatic effects was -3.5±11.3d, indicating also the mechanism of homeostasis.

## Discussion

### Genetic mechanisms of QTLs on heading date

Heading date is one of complex quantitative traits controlled by a multiple gene system^3,7^. At least more than 734 heading date QTLs were identified in rice (http://archive.gramene.org/qtl/). Four QTLs, *Hd1*, *Ehd1*, *OsMADS50* and *Hd3a*, have detailed gene products and biological functions^4,12,13,15,30,31^. The interactions among the four QTLs have also been explored in-depth via molecular technique^6,32,33^, and preliminary genetic networks have also been formed for rice flowering^34^. In one of our previous papers, we tested the phenotypic functions for the four QTLs and their epistatic effects between dual QTLs, and confirmed also the existing of flowering network^28^. In this paper, the four QTLs on heading date were tested again in three seasons of two years (Table 1 and Table 2). We detected that *Ehd1* delayed heading under the status of homozygote or heterozygote, which was regulated by environmental conditions. *OsMADS50* and *Hd3a* always promoted and delayed heading, respectively. *Hd1* had a little effect on heading date. We also found the network relationship of the four QTLs. *Hd1* and *Ehd1* were independent, while the other QTLs related each other. *Hd1* and *Ehd1* regulated flowering via to directly or indirectly regulate *Hd3a* to form two flowering pathways (Fig. 1). These results basically were consistent with those in the previous study^28^. However, *OsMADS50* also directly or indirectly regulated *Hd3a* than to influence flowering, perhaps being a new flowering path.

### Epistasis among QTLs on heading date

Epistasis is an important genetic component and a plausible feature of the genetic architecture of quantitative traits^20,26^. Epistatic interactions between QTLs on heading date were found qualitatively in earlier studies^4,12,15–17^, and were quantificationally estimated based on near isogenic lines and SSSLs^18,24,25^. Utilization of SSSLs, we estimated epistatic components between dual QTLs on lots of important traits such as tiller number, plant height, heading date, yield and its component traits^5,28,29,35–42^. One common feature for epistasis was verified again by this paper, i.e. less-than-additive epistatic interactions of quantitative trait loci^24^. Here 75% of QTL effects were positive (Table 1 and Table 2), then 62.5% of epistatic components between two QTLs appeared negative (Table 4). In fact, epistatic effects estimated would be mostly negative if two QTL effects were positive^29,41^. The change of a gene effect may be brought about by modification of gene function due to alterations in the signal-transducing pathway^21^. Opposite expression between genes and gene interactions was considered to be an important mechanism for maintaining homeostasis^27^. That mixed epistatic effects in a genotype of triple QTLs were always opposite with the sum of single gene effects also showed the mechanism (Table 6). The combination of three positive effect QTLs usually generated negative mixed epistatic effect.

### Application of gene interactions on heading date

Knowledge of epistatic interactions not only improve our understanding of genetic networks and mechanisms that underlie genetic homeostasis, but also enhance predictions of responses to artificial pyramiding breeding for quantitative traits in agricultural crop species. The success of molecular pyramiding breeding dependents directly on gene interactions except for gene additive. Line breeding is to select improved homozygous genotypes, in which additive and additive × additive epistasis play a leading role. Like the additive effect, the epistasis of additive × additive is also a stabilize genetic component between selfing generations. Full consideration of additive × additive epistasis is helpful to evaluate the potential benefits of special combining ability in breeding^43^. In this paper, *OsMADS50* seemed to have large and negative average effects of interactions with other genes (Table 4), this QTL can be applied to early ripe breeding. Reversely, *Hd3a-2* always generated large and positive epistatic effects, this gene can be acted as pyramiding material for late ripe breeding. For the interactions among three QTLs, we should consider simultaneously the dual-gene interaction and the triple gene interaction, i.e. mixed epistasis (Table 6). Since both *OsMADS50-2* and *Hd1-2* had the mixed epistatic effects to promote heading in this paper, which are perhaps appropriate genes for the pyramiding breeding of early ripe rice. That Hd3a-2 mostly generated large and positive mixed epistatic effects also indicated that this gene can be applied to late ripe breeding in rice.

Heterosis is a very common phenomenon in plant breeding, and was deciphered by many hypotheses. The dominant hypothesis and the superdominant hypothesis were the most representative^44^. However, these hypotheses were based on single-gene theory. Multiple gene theory suggested that heterosis was closely related to epistatic effects except for dominant effect^45^. In this paper, we detected four dominant QTLs on heading date, all of which appeared superdominant effects (Table 1 and Table 2). These dominant QTLs would generate three types of dual gene interactions (Table 4) and seven types of triple gene interactions (Table 5). Compared with additive-additive epistatic components (3.2±7.3d), the dominance-dominance epistatic components (-8.2±7.2d) were always small. Obviously, the dominance and its epistasis play a greater role than the additive and its epistasis between QTLs for breeding of early ripe varieties. Thus, the heterozygote had often greater advantage in early ripe than the homozygote between two QTLs. For triple QTL combinations, the case was the same. The mixed epistatic component of additive-additive-additive was 4.8±8.2d, while dominance- dominance-dominance was -12.5±6.6d. It also showed that the heterozygote of triple QTLs still appeared heterosis in early ripe since the mixed epistatic components. Thus, we suggested that the dominance and its epistasis of QTLs were the main genetic factors to result in heterosis of early ripening.

In practice, when an epistatic effect was not significant or with the same direction as the effects of constituted QTLs, these QTLs might be considered as gene materials for molecular breeding^28^. In this paper, negative effect *OsMADS50* and positive effect *Hd3a-2* generated always the same direction effects of dual QTL epistasis and triple QTL mixed epistasis, they might be the ideal gene materials for different breeding objectives.

## Conclusion

Four rice SSSLs were identified to be loaded with heading date QTLs *Hd1, Ehd1, OsMADS50* and *Hd3a*, respectively. There were 49 and 4 out of 56 dual QTL and triple QTL pyramiding materials to have significant effects in all environments and only in special environments, respectively. *Hd3a* is the flowering factor, which were regulated by the other three QTLs to influence heading. The four QTLs formed at least 4 flowering paths. All of six pairs of tested QTLs generated interaction, 18 of 24 epistatic components were significant, and 10 out of 18 significant epistases were negative. 81.25% (out of 32) interactions of triple QTLs were significant, and 57.7% (out of 26) significant epistasis were positive. 90.6% (out of 32) mixed epistases were significant, and 66.7% (out of 27) significant epistases were negative. The relationship “positive QTLs-negative one order interactions-positive two order interactions” indicated that the aggregation effect of QTLs was partially neutralized by the opposite epistatic effect sum. Epistasis played a role of homeostasis. *OsMADS50* was suitable to early ripe breeding, while *Hd3a-2* to late ripe breeding. The results indicated that QTL epistasis plays a role of homeostasis on heading date in rice.

## Materials and methods

### Plant materials

**Figure 2:**
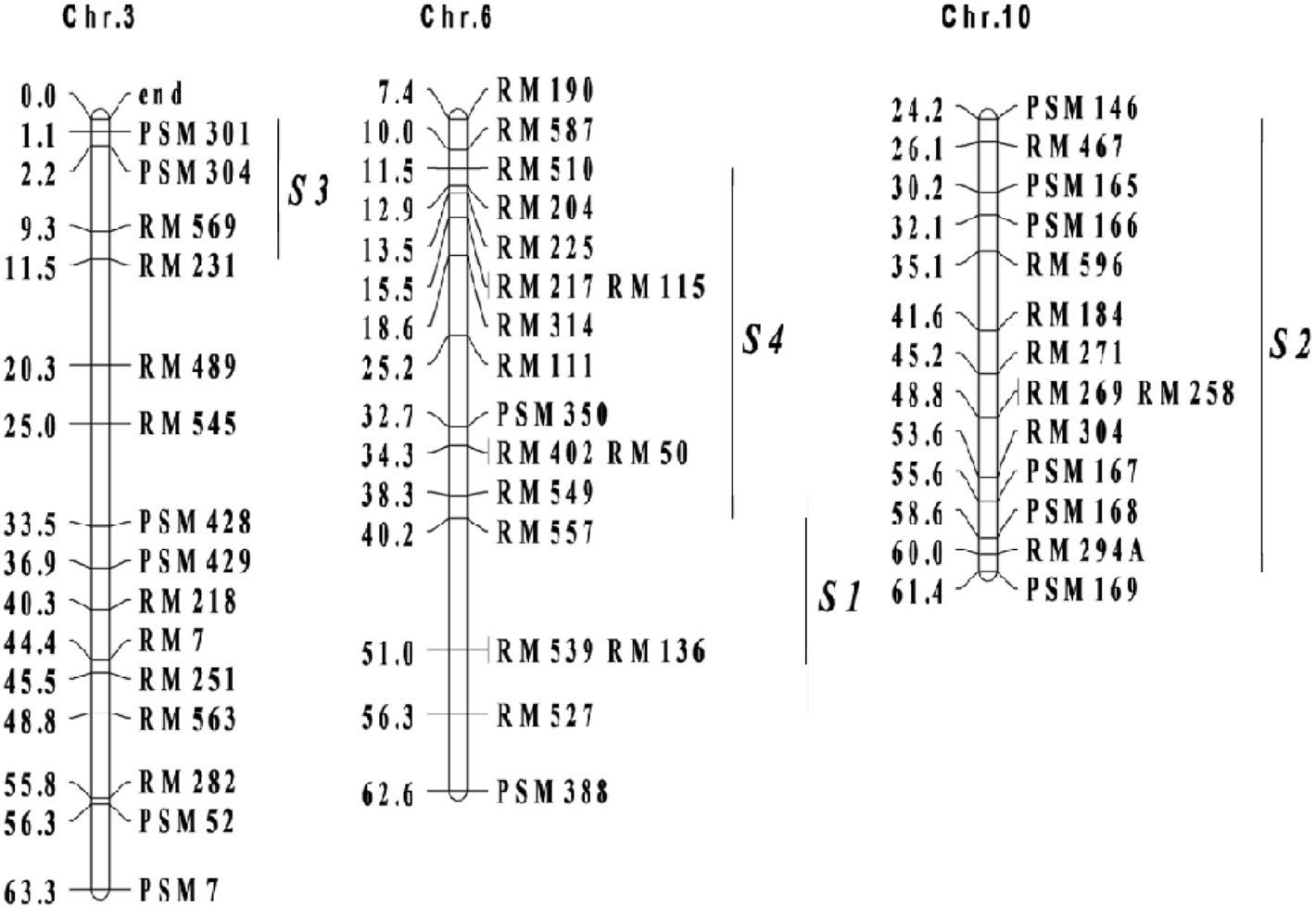
Marker intervals for substitution segments of single segment substitution lines on corresponding chromosomes. Chr. and *S* were the abbreviation of chromosome and single segment substitution line, followed by serial numbers, respectively. The rectangular frames and the bold vertical lines represented chromosomes and substitution segments from donors of single segment substitution lines, respectively. The genetic distances (cM) for each marker and the marker names were listed on either side of chromosomes, respectively.

Hua-jing-xian 74 (HJX74) and its four single segment substitutions lines (SSSLs) were as basic experimental materials. HJX74 is an elite indica variety, developed by our laboratory, Guangdong Key Laboratory of Plant Molecular Breeding at South China Agricultural University in Guangzhou of China. SSSL contains only one segment of donor chromosome introgressed into a recipient genetic background. A SSSL library, nearly 2000 members, was bred by successive backcrosses, in which HJX 74 was as the recipient parent and more than 30 excellent varieties from around the world as the donor parents^46^. Relevant SSR markers were applied to foreground selection of donor segments and background selection of HJX74^47,48^. In our previous studies the 4 SSSLs were detected with QTLs controlling heading date in rice, and then epistases between double QTLs were analyzed via pyramiding of SSSLs^28^. Some background information for SSSLs, including the SSSL codes, heading date QTL names, donor varieties, and marker intervals on corresponding chromosomes, were showed in Table 7 and Fig. 2, respectively.

**Table 7:** The codes, heading date QTLs and donor sources of single segment substitution lines (SSSLs).

SSSL	Code	QTL on heading date	Donor source
$S_{1}$	W08-18-09-09-06-02	$Hd1$	IR64
$S_{2}$	W27-18-03-21	$Ehd1$	IAPAR9
$S_{3}$	W23-03-08-09-27-82	$OsMADS50$	Lemont
$S_{4}$	W04-47-68-05-04-04-02-02	$Hd3a$	BG367

Some pyramiding materials of SSSLs (including homozygotes and heterozygotes) were configured to analyze epistasis among QTLs. The crossing between a SSSL and HJX74 would generate the heterozygote of SSSL. From the *F*_*2*_ populations derived from the *F*_*1*_ crossing combinations between two SSSLs, the homozygotes and the heterozygotes of dual QTLs could be obtained by marker assisted selection. Similarly, triple-QTL pyramiding materials could also be selected from the *F*_*2*_ generation of three QTL combinations.

### Field trials

Phenotypic experiments were conducted at the experimental farm of South China Agricultural University, Guangzhou (at ~ 113° east longitude and ~ 23° north latitude), China, in spring (from March to July, suggested as natural long-day condition, NLD) 2016 and autumn (from July to November, suggested as natural short-day condition, NSD) 2015 and 2016, respectively. Meteorological data showed that the average duration of possible sunshine is larger than 13 h under the spring season and less than 12 h under the autumn season in Guangzhou. A total of 65 plant materials, including HJX74, 4 homozygotes and 4 heterozygotes of SSSLs, 24 dual-QTL polymers and 32 triple-QTL polymers, were grown in all three environments. In each experiment, the germinated seeds were sown in a seedling bed and seedlings were transplanted to a paddy field 20 days later, with one plant per hill spaced at 16.7 cm × 16.7 cm. A randomized block design was adopted in field trails, in which each plot consisted of four rows with ten plants each row. The management of the field experiments was in accordance with local standard practices. The heading date (HD) of twenty plants at the center of each plot was measured as the number of days from sowing to the appearance of the first panicle. Averages on HD over twenty plants each plot were as inputting data for statistical analysis.

### Mixed linear models for estimating G effects and GE interaction effects

For a genetic experiment conducted within multiple environments, the phenotypic performance of the jth genetic entry in the kth block within the hth environment can be expressed by,

$$ y_{hjk} = \mu + E_{h} + G_{j} + GE_{hj} + B_{k/h} + e_{hjk} $$

where $y, \mu , E, G, GE, B$ and $e$ were the observation value each plot, population mean value, environmental effect, genotypic effect, genotype-environment interaction effect, block effect and the residual error, respectively. The minimum norm quadratic unbiased estimation (MINQUE) method with all prior values set at 1 was used to estimate variance components for the trait^44^. Values of *G* and *GE* were predicted by the Best Linear Unbiased Prediction (BLUP) method^44^. All estimations were performed using the QGAStation software package^49^.

### QTL analysis

An indirect approach was conducted to analyze QTL effects^50^. First, values of G and GE for all genetic materials on HD were estimated according to the model mentioned above, respectively. Next, QTLs were mapped using these estimated values as input data separately. QTLs identified according to G were referred to main QTLs, including additive effect (*a*), dominant effect (*d*) and epistatic effect (*e*). QTLs obtained from GE were called as interaction QTLs, including additive interacted by environment (*ae*), dominance interacted by environment (*de*), and epistasis interacted by environment (*ee*). According to the models $G = a + d + e$ and $GE = ae + de + ee$ the effect values of QTLs could be estimated by the lm( ) function in R language(http://cran.r-project.org).

### Experimental research

Experimental research and field studies on plants (either cultivated or wild), including the collection of plant material, must comply with relevant institutional, national, and international guidelines and legislation.

### Supplementary Information

Supplementary Information 1.

Supplementary Information 2.

## Data Availability

All data generated or analysed during this study are included in this published article and its supplementary information file Supp.zip.
