# Supplementary material for: QTL epistasis plays a role of homeostasis on heading date in rice
Source: Sci Rep. 2024 Jan 3;14:373. doi: 10.1038/s41598-023-50786-x (PMC10764746; doi:10.1038/s41598-023-50786-x)
Supplement: Supplementary file 1 — Supplementary Information 1. [file 41598_2023_50786_MOESM1_ESM.docx]

Figure S1 The effect changes of 4 QTLs under different background QTLs. Background QTLs mean the QTLs combined with the target QTL. For instance, *Ehd1-1* is the target QTL, *OsMADS50-1* is the background QTL in the combination of *Ehd1-1/OsMADS50-1,* while *Hd3a-1* is the background QTL in the combination of *Ehd1-1/Hd3a-1.*
