# Supplementary material for: QTL epistasis plays a role of homeostasis on heading date in rice
Source: Sci Rep. 2024 Jan 3;14:373. doi: 10.1038/s41598-023-50786-x (PMC10764746; doi:10.1038/s41598-023-50786-x)
Supplement: Supplementary file 2 — Supplementary Information 2. [file 41598_2023_50786_MOESM2_ESM.docx]

Table S1 Analysis of variance on phenotypic values of heading date in the three environments. $\sigma_{e}^{2},\sigma_{G}^{2},\sigma_{E}^{2},\sigma_{GE}^{2}$were the variance components of error, genotype, environment and genotype$\times$environment, respectively. $r,k,h$ represented the numbers of replication, genotype and environment, respectively.

| Source | DF | Sum square | Mean square | F value | Pr>F | EMS |
| --- | --- | --- | --- | --- | --- | --- |
| Environment | 2 | 60726.66 | 30363.33 | 14659.60 | <0.0001 | $\sigma_{e}^{2}+r\sigma_{GE}^{2}+kr\sigma_{E}^{2}$ |
| Genotype | 64 | 43828.74 | 684.82 | 330.64 | <0.0001 | $\sigma_{e}^{2}+r\sigma_{GE}^{2}+hr\sigma_{G}^{2}$ |
| Genotype x Environment | 128 | 1411.62 | 11.03 | 5.32 | <0.0001 | $\sigma_{e}^{2}+r\sigma_{GE}^{2}$ |
| Error | 374 | 774.64 | 2.07 |  |  | $\sigma_{e}^{2}$ |
| Corrected Total | 568 | 106741.65 |  |  |  |  |
